# *N*-acetylglucosaminyltransferase II Is Involved in Plant Growth and Development Under Stress Conditions

**DOI:** 10.3389/fpls.2021.761064

**Published:** 2021-11-02

**Authors:** Jae Yong Yoo, Ki Seong Ko, Bich Ngoc Vu, Young Eun Lee, Seok Han Yoon, Thao Thi Pham, Ji-Yeon Kim, Jae-Min Lim, Yang Jae Kang, Jong Chan Hong, Kyun Oh Lee

**Affiliations:** ^1^Plant Molecular Biology and Biotechnology Research Center (PMBBRC), Jinju, South Korea; ^2^Division of Applied Life Sciences (BK4 Program), Jinju, South Korea; ^3^Department of Chemistry, Changwon National University, Changwon, South Korea; ^4^Division of Life Science, Jinju, South Korea; ^5^Division of Bio & Medical Bigdata (BK4 Program), Gyeongsang National University, Jinju, South Korea

**Keywords:** GnTII, N-glycan, structural diversity, functional diversity, plant development, stress response

## Abstract

Alpha-1,6-mannosyl-glycoprotein 2-β-*N*-acetylglucosaminyltransferase [EC 2.4.1.143, *N*-acetylglucosaminyltransferase II (GnTII)] catalyzes the transfer of N-acetylglucosamine (GlcNAc) residue from the nucleotide sugar donor UDP-GlcNAc to the α1,6-mannose residue of the di-antennary N-glycan acceptor GlcNAc(Xyl)Man_3_(Fuc)GlcNAc_2_ in the Golgi apparatus. Although the formation of the GlcNAc_2_(Xyl)Man_3_(Fuc)GlcNAc_2_ N-glycan is known to be associated with GnTII activity in *Arabidopsis thaliana*, its physiological significance is still not fully understood in plants. To address the physiological importance of the GlcNAc_2_(Xyl)Man_3_(Fuc)GlcNAc_2_ N-glycan, we examined the phenotypic effects of loss-of-function mutations in *GnTII* in the presence and absence of stress, and responsiveness to phytohormones. Prolonged stress induced by tunicamycin (TM) or sodium chloride (NaCl) treatment increased *GnTII* expression in wild-type Arabidopsis (ecotype Col-0) but caused severe developmental damage in GnTII loss-of-function mutants (*gnt2-1* and *gnt2-2*). The absence of the 6-arm GlcNAc residue in the N-glycans in *gnt2-1* facilitated the TM-induced unfolded protein response, accelerated dark-induced leaf senescence, and reduced cytokinin signaling, as well as susceptibility to cytokinin-induced root growth inhibition. Furthermore, *gnt2-1* and *gnt2-2* seedlings exhibited enhanced N-1-naphthylphthalamic acid-induced inhibition of tropic growth and development. Thus, GnTII’s promotion of the 6-arm GlcNAc addition to N-glycans is important for plant growth and development under stress conditions, possibly *via* affecting glycoprotein folding and/or distribution.

## Introduction

Proteins destined for secretion or incorporation into cellular membranes are initially transferred into the endoplasmic reticulum (ER) as they are being translated on membrane-bound ribosomes (Gomord and Faye, 2004; Walsh, 2010). Attachment of the oligosaccharide known as glycan to the asparagine residue within the consensus sequence Asn-X-Ser/Thr (where X is any amino acid but proline) during the translocation of the nascent polypeptide into the ER is a common and highly conserved protein modification in eukaryotes, although it is rare in prokaryotes (Wilson, 2002; Faye et al., 2005; Gomord et al., 2010). In plants, the majority of extracellular and transmembrane proteins, including hormone receptors and transporters, is glycosylated at multiple positions *via* N-linked oligosaccharides (Hwang and Sheen, 2001; Vavra et al., 2017). As one of the most significant posttranslational modifications, N-glycosylation has a substantial effect on the physicochemical properties and biological functions of a variety of glycoproteins in plants (Hong et al., 2009; Fanata et al., 2013; Harmoko et al., 2016). Given the diversity of substrates for glycosyltransferase and glycosidase, a genetic or epigenetic variation may have a pleiotropic influence on plant development. N-glycosylation plays a role in regulating the folding and stability of proteins, their distribution to specific subcellular locations, their interaction with lectins or other proteins, and their activities and signaling functions in eukaryotic cells (Hebert et al., 2014).

N-glycosylation commences with the biosynthesis of lipid-linked oligosaccharides, which begins on the cytoplasmic side and is completed on the luminal side of the ER membrane. This process is carried out by the serial reactions of glycosyltransferases that are encoded by the asparagine-linked glycosylation (*ALG*) genes (Heesen et al., 1994; Aebi et al., 1996; Burda et al., 1996; Colussi et al., 1997; Burda and Aebi, 1998; Burda et al., 1999; Gao et al., 2004; Noffz et al., 2009). TM inhibits the early phase of N-glycosylation, resulting in an increased accumulation of unfolded protein in the ER lumen and activation of the UPR (Liu et al., 2007a; Mishiba et al., 2013). Subsequently, the oligosaccharyltransferase complex transfers the 14-sugar oligosaccharide precursor (Glc_3_Man_9_GlcNAc_2_) *en bloc* from the dolichol lipid carrier to an Asn residue within the N-glycosylation consensus sequence of a nascent polypeptide in the ER (Gallois et al., 1997; Lerouxel et al., 2005; Kelleher and Gilmore, 2006; Ruiz-Canada et al., 2009). The initial trimming of the oligosaccharide precursor occurs in the ER, and then, the glycoprotein is trafficked to the Golgi, where it is further modified and processed (Hebert et al., 2005). N-glycosylation is completed in the *trans*-Golgi network, and further trimming of N-glycans may take place at the plasma membrane and in the vacuole (Lerouge et al., 1998; Strasser, 2016).

While the early N-glycosylation processes in the ER and *cis*-Golgi are highly conserved among vertebrates and plant species, the later steps that form complex N-glycans in the medial and *trans* compartments of the Golgi stacks are divergent among species (Puccia et al., 1993; Gonzalez et al., 1999; Liebminger et al., 2009; Schwarz and Aebi, 2011). The GlcNAcMan_3_GlcNAc_2_ structure produced by *N*-acetylgl ucosaminyltransferase I (GnTI) and Golgi α-mannosidase II (GMII) in the plant Golgi serves as a common acceptor of GlcNAc, xylose, and fucose residues through the activities of *N*-acetylglucosaminyltransferase II (GnTII), β1,2-xylosyl transferase (XylT), and α1,3-fucosyltransferase (FucT), respectively (Kang et al., 2008; Schoberer and Strasser, 2011; Yoo et al., 2015). Both limited addition of the 6-arm GlcNAc residue by GnTII and processing of the 3-arm GlcNAc residue by β-*N*-acetylhexosaminidases (HEXOI, II, and III) facilitate formation of the paucimannose N-glycan [(Xyl)Man_3_(Fuc)GlcNAc_2_] in plants (Liebminger et al., 2011; Yoo et al., 2015). Thus, plant cells mainly produce the paucimannose N-glycan with core β1,2-xylose and α1,3-fucose residues at the first mannose residue of the trimannosyl core and innermost GlcNAc residue, respectively.

The biosynthesis of the N-glycan precursor and its transfer to a nascent protein play important roles in plant development and stress tolerance. *Arabidopsis thaliana* mutants of the Golgi-localized N-glycan processing enzymes, such as the *cgl1* (harboring a defect in *GnTI*), *hgl1* (harboring a defect in *GMII*), *xylt* (*XylT*), and *fucta* and *fuctb* (*FucTa* and *FucTb*), do not display obvious phenotypic abnormalities under normal growth conditions but are more sensitive to stress than are their wild-type siblings (Kang et al., 2008; Yoo et al., 2015). By contrast, rice (*Oryza sativa*) *gnt1* mutants that fail to produce complex N-glycans show defective post-seedling development and early lethality without transition to the reproductive stage (Fanata et al., 2013). Rice *fuct-1* and *fuct-2* mutants that produce N-glycans lacking α1,3-fucose residues display a larger tiller angle, shorter internode and panicle lengths, and decreased grain filling rate as well as chalky grains with abnormal shape (Harmoko et al., 2016). Previous results indicate that further processing of N-glycan in the Golgi apparatus is associated with the stress response and that the effects of the Golgi N-glycan processing on plant development are dependent on N-glycan structure and plant species (Kang et al., 2008; Fanata et al., 2013; Harmoko et al., 2016).

Previously, we proposed that both selective trimming of the 3-arm nonreducing β1,2-GlcNAc residue and limited addition of the 6-arm nonreducing β1,2-GlcNAc residue, executed by HEXOI, II, and III and by GnTII, respectively, facilitate the formation of the major (Xyl)Man_3_(Fuc)GlcNAc_2_ and a minor GlcNAc_2_(Xyl)Man_3_(Fuc)GlcNAc_2_ N-glycan structures in plants (Yoo et al., 2015). However, the physiological significance of the minor N-glycan that is associated with GnTII activity is still not fully understood in plants. Humans with point mutations in the catalytic domain of *Mgat2*, which encodes GnTII, have a congenital disorder of glycosylation type IIa, a syndrome characterized by a general failure to thrive, dysmorphic facial features, feeding difficulties, and psychomotor retardation (Jaeken et al., 1993, 1994; Tan et al., 1996). A mutant mouse with a deletion of *Mgat2* was deficient in GnTII activity and complex N-glycan biosynthesis, resulting in severe gastrointestinal, hematologic, and osteogenic abnormalities (Wang et al., 2001, 2002). Targeted disruption of the *Fusarium oxysporum* GnTII gene results in altered cell wall properties and a dramatic reduction in virulence against both plant and animal hosts (Lopez-Fernandez et al., 2013). In this study, we compared the developmental features of Arabidopsis T-DNA insertion mutants (*gnt2-1* and *gnt2-2*), which lack GnTII activity, with their respective wild-types Col-0 or Ws. We also analyzed the distinctive phenotypes of the *gnt2-1* and *gnt2-2* plants in response to stresses and phytohormones to gain insight into the physiological functions of the minor N-glycan in plants.

## Results

### *GnTII* Expression Is Associated With Development and Stress Responses in Arabidopsis

**Figure 1:**
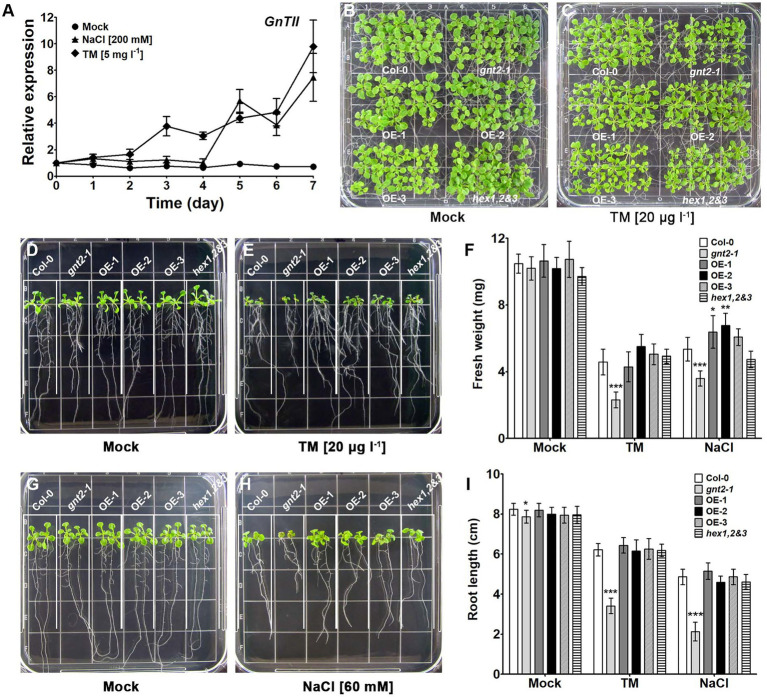
Stress-induced gene expression is associated with developmental defects in the loss-of-function mutant of GnTII (*gnt2-1*) under stress conditions. **(A)** Expression of *GnTII* in 10-day-old Arabidopsis Col-0 seedlings treated with MS medium alone (mock treatment) or supplemented with 200mm NaCl or 5mgl^−1^ TM for 7day analyzed by qPCR. The experiment was performed independently three times (three biological replicates) and normalized with respect to *β-TUBULIN* expression. Data represent means±SEM of the three independent experiments. **(B-I)** Col-0, *gnt2-1*, OE-1, OE-2, OE-3, and *hex1,2&3* plants were grown for 12day, horizontally **(B,C)** or vertically **(D,E,G,H)**, on MS plates **(B,D,G)** or MS plates containing 20μgl^−1^ TM **(C,E)** or 60mm NaCl **(H)**, respectively. The grid on the square plate is 18×18mm. Photographs were taken 12day after plating, and the fresh weight **(F)** and root length **(I)** were measured. Asterisks indicate statistically significant differences (^*^p<0.05, ^**^p<0.01, and ^***^p<0.001) as determined by two-way ANOVA.

Previous findings indicated that the regulated activities of the β-hexosaminidases and GnTII contribute to the diversity of N-glycan structures, facilitating the formation of the major paucimannose N-glycan, (Xyl)Man_3_(Fuc)GlcNAc_2_, and the minor complex N-glycan, GlcNAc_2_(Xyl)Man_3_(Fuc)GlcNAc_2_, in the Golgi apparatus of plant cells (Yoo et al., 2015). This prompted us to investigate in more detail whether the structural diversity of N-glycans is necessary and whether the minor complex N-glycan plays an important role in plant cells. Since complex N-glycans played important roles in the stress response and tolerance of Arabidopsis (Kang et al., 2008), we wondered whether *GnTII* expression is associated with plant growth and development under stress conditions. It seems likely that *GnTII* expression can be increased under stress to have a positive effect on stress tolerance and stress response in Arabidopsis. We therefore treated Col-0 plants with either MS medium (mock treatment) or MS medium supplemented with NaCl or TM and analyzed *GnTII* transcription by qPCR to investigate how it might be altered under stress conditions. While *GnTII* transcription did not increase in response to short-term stress, we found that it did increase in response to prolonged stress (Figure 1A). This finding suggests that GnTII can play an important role in mediating chronic stress responses.

Since *GnTII* expression was induced in response to chronic stress, we wondered whether the minor complex N-glycan, GlcNAc_2_Man_3_(Fuc)GlcNAc_2_, which is produced in a GnTII-dependent manner, plays an important role in the plant’s response to prolonged stress. We therefore used the Col-0, *gnt2-1* mutants, transgenic Arabidopsis plants overexpressing GnTII (three independent lines OE-1, OE-2, and OE-3), and *hex1,2&3* plants lacking β-N-hexosaminidase activity to assess the physiological importance of the minor complex N-glycan under chronic stress (Supplementary Figure S1A). When the plants were grown on MS medium, *gnt2-1*, OE-1, OE-2, OE-3, and *hex1,2&3* seedlings did not significantly differ from Col-0 seedlings in the phenotypic characteristics we assessed, which included fresh weight and root length (Figures 1B,D,F,G,I). However, when cultured on MS medium containing 60mm NaCl or 20μgl^−1^ TM, *gnt2-1* seedlings, but not OE-1, OE-2, OE-3 or *hex1,2&3* seedlings, showed significant decreases in fresh weight and root length compared to Col-0 seedlings (Figures 1C,E,F,H,I). This finding suggests that the GnTII-based minor complex N-glycan plays a role in plant growth and development under prolonged stress.

### The Minor Complex N-glycan, GlcNAc_2_(Xyl)Man_3_(Fuc)GlcNAc_2_, Is Missing in *gnt2-1*

To confirm whether the stress-sensitive phenotype of *gnt2-1* under chronic stress is caused by altered structure or diversity of N-glycans, immunoblot and lectin blot analyses were performed on proteins extracted from Col-0, *gnt2-1*, OE-1, and *hex1,2&3* plants grown in the presence or absence of TM to (Supplementary Figure S2). Anti-HRP, a polyclonal antibody previously shown to bind peptides with N-glycans containing α1,3-fucose and/or β1,2-xylose residues (Fanata et al., 2013; Harmoko et al., 2016), anti-fucose, and anti-xylose antibodies interacted with proteins extracted from *gnt2-1* in a different pattern compared with proteins extracted from Col-0. This result is consistent with previous findings that α1,3-fucosyltransferase, β1,2-xylosyltransferase and GnTII interact competitively with the GlcNacMan_3_Glc_2_ N-glycan in plant medial Golgi (Yoo et al., 2015). However, these antibodies interacted less with proteins extracted from *hex1,2&3*. The interactions of these antibodies with proteins extracted from OE-1 plants were comparable to those with proteins extracted from Col-0 plants. No differences in the interactions of proteins extracted from Col-0, *gnt2-1*, OE-1, and *hex1,2&3* plants with ConA and GSII were found. There were no differences in antibody interactions between proteins extracted from plants grown in the presence or absence of TM in our experiments (Supplementary Figure S2).

To obtain more information on the detailed N-glycan structures in Col-0, *gnt2-1*, OE-1, and *hex1,2&3* plants, we analyzed the N-glycans released by peptide:N-glycosidase A treatment of proteins from the four genotypes of plants using an Orbitrap-based mass spectrometer. The N-glycan profiles revealed by the mass spectrometry (MS) data indicated that Col-0 plants produce both the major (Xyl)Man_3_(Fuc)GlcNAc_2_ N-glycan form and the minor GlcNAc_2_(Xyl)Man_3_(Fuc)GlcNAc_2_ N-glycan, while the *gnt2-1* plants produce the major (Xyl)Man_3_(Fuc)GlcNAc_2_ paucimannose N-glycan but not the minor GlcNAc_2_(Xyl)Man_3_(Fuc)GlcNAc_2_ complex N-glycan (Supplementary Figures S3–S6). The relative amount of the GlcNAc_2_(Xyl)Man_3_(Fuc)GlcNAc_2_ complex N-glycan was higher in proteins extracted from OE-1 plants than in Col-0 plants. In the *hex1,2&3* plants, the GlcNAc_2_(Xyl)Man_3_(Fuc)GlcNAc_2_ complex N-glycan was the most abundant, while the paucimannose (Xyl)Man_3_(Fuc)GlcNAc_2_ was almost undetectable (Supplementary Figures S3–S6). Anti-HRP, anti-fucose, and anti-xylose antibodies interacted weakly with *hex1.2&3* plant proteins, while MS analysis showed that the most abundant N-glycan is GlcNAc_2_(Xyl)Man_3_(Fuc)GlcNAc_2_. According to these findings, anti-HRP, anti-fucose, and anti-xylose antibodies should have a lower affinity for antigens containing the GlcNAc_2_(Xyl)Man_3_(Fuc)GlcNAc_2_ complex N-glycan than for antigens containing the (Xyl)Man_3_(Fuc)GlcNAc_2_ paucimannose N-glycan (Supplementary Figures S2–S6). This finding indicates that the *gnt2-1* mutant lacks the minor complex N-glycan which could be linked to the phenotype of abnormal growth in Arabidopsis under prolonged stress.

### GnTII Loss-of-Function Mutation Enhances the Punctate Pattern of Callose Deposition During Prolonged Stress

**Figure 2:**
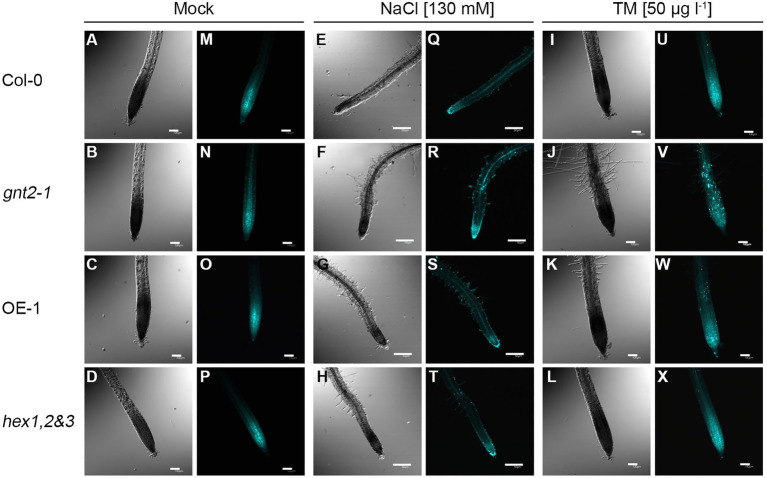
The *gnt2-1* mutant enhances the punctate pattern of callose deposition under prolonged stress conditions. *In situ* callose deposition at the root tip was visualized by aniline blue staining and viewed by fluorescence microscopy. **(A–X)** Five-day-old Col-0, *gnt2-1*, OE-1, and *hex1,2&3* plants were further grown on MS medium **(A–D, M–P)**, MS medium supplemented with 130mm NaCl **(E–H, Q–T)** or MS medium supplemented with 50μgl^−1^ TM **(I–L, U–X)** for 4day, respectively, and stained with 0.005% discolorized aniline blue. The punctate pattern of callose deposition was visualized by using a fluorescent microscope equipped with a DAPI filter set. Notice the strong punctate callose deposition in *gnt2-1* plants under prolonged stress conditions **(R,V)**. Scale bar in mock and TM treatment=100μm, scale bar in NaCl treatment=50μm.

Callose (β-1,3-glucan) is a key regulator of plasmodesmata permeability as well as a key player in plant growth, development, and stress responses (Jacobs et al., 2003; Cui and Lee, 2016). Since the *gnt2-1* mutant showed severe developmental defects, including decreases in fresh weight and root length, we wondered whether the loss-of-function mutation in *GnTII* is associated with aberrant callose deposition during prolonged stress. To answer this question, we compared the pattern of callose deposition in the plant root apices of Col-0, *gnt2-1*, OE-1, and *hex1,2&3* plants grown on standard MS medium vs. MS medium containing 130mm NaCl or 50μgl^−1^ TM and (Figure 2). When the plants were cultured on MS medium, none of the four plants—Col-0, *gnt2-1*, OE-1, and *hex1,2&3*—showed significant differences in callose deposition in the root apices (Figures 2M–P). However, when grown on MS medium supplemented with 130mm NaCl or 50μgl^−1^ TM, *gnt2-1* plants, but not OE-1 or *hex1,2&3* plants, exhibited a distinct punctate pattern of callose deposition in the root apices (Figures 2R,V) compared to Col-0 plants (Figures 2Q,S–U,W,X). This result indicates that the minor complex N-glycan plays a role in root growth and stress responses by regulating callose deposition in root apices during prolonged stress.

### GnTII Loss-of-Function Mutation Enhances UPR During Prolonged Stress

**Figure 3:**
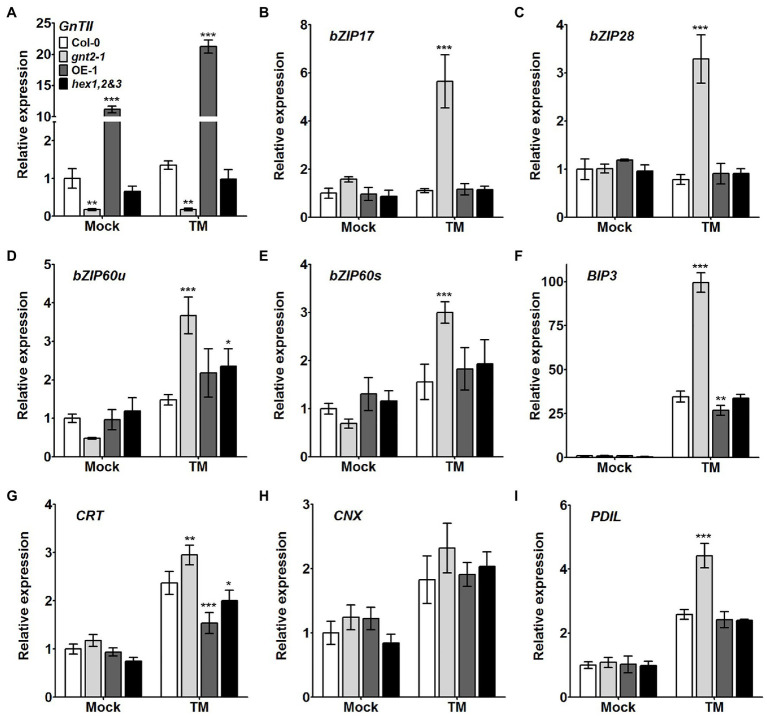
Expression of UPR-related genes is higher in the *gnt2-1* mutant than in Col-0 plants under prolonged ER stress. **(A)** Analysis of *GnTII* expression in Col-0, *gnt2-1*, OE-1, and *hex1,2&3* plants. **(B–I)** Relative expression of mRNAs encoding various UPR-related genes in Col-0, *gnt2-1*, OE-1, and *hex1,2&3* plants analyzed by qPCR. Total RNA was extracted from 12-day-old seedlings grown on MS medium with or without TM (20μgl^−1^). The experiment was performed independently three times (three biological replicates) and normalized with respect to *β-TUBULIN* expression. Data represent mean±SEM of three independent experiments. Asterisks indicate statistically significant differences (^*^p<0.05, ^**^p<0.01, and ^***^p<0.001) as determined by two-way ANOVA.

Because expression of *GnTII* was induced by salt stress and ER stress, and *gnt2-1* was more responsive to these two forms of stress than Col-0, we hypothesized that the *GnTII* loss-of-function mutation would result in an enhanced UPR under prolonged stress. Thus, we examined expression of UPR-related genes, such as *bZIP17*, *bZIP28*, *bZIP60u*, *bZIP60s*, *BIP3*, *CRT*, *CNX*, and *PIDL*, in Col-0, *gnt2-1*, OE-1, and *hex1,2&3* plants grown on standard MS medium or MS medium containing TM (Figure 3). When the plants were grown on MS medium, there were no significant differences in the expression of the UPR genes between Col-0, *gnt2-1*, OE-1, and *hex1,2&3* plants (Figure 3). However, when the plants were grown in the presence of 20μgl^−1^ TM, UPR gene expression was significantly higher in *gnt2-1* plants than in Col-0, but not in OE-1 or *hex1,2&3* plants (Figure 3). Thus, the stress-sensitive phenotype of *gnt2-1* is associated with UPR activation by prolonged stress (Figures 1B–I). It is possible that the lack of the minor complex N-glycan results in an increase in unfolded proteins, which in turn causes *gnt2-1* to have a higher UPR under prolonged stress.

### GnTII Loss-of-Function Mutation Facilitates Dark-Induced Leaf Senescence

**Figure 4:**
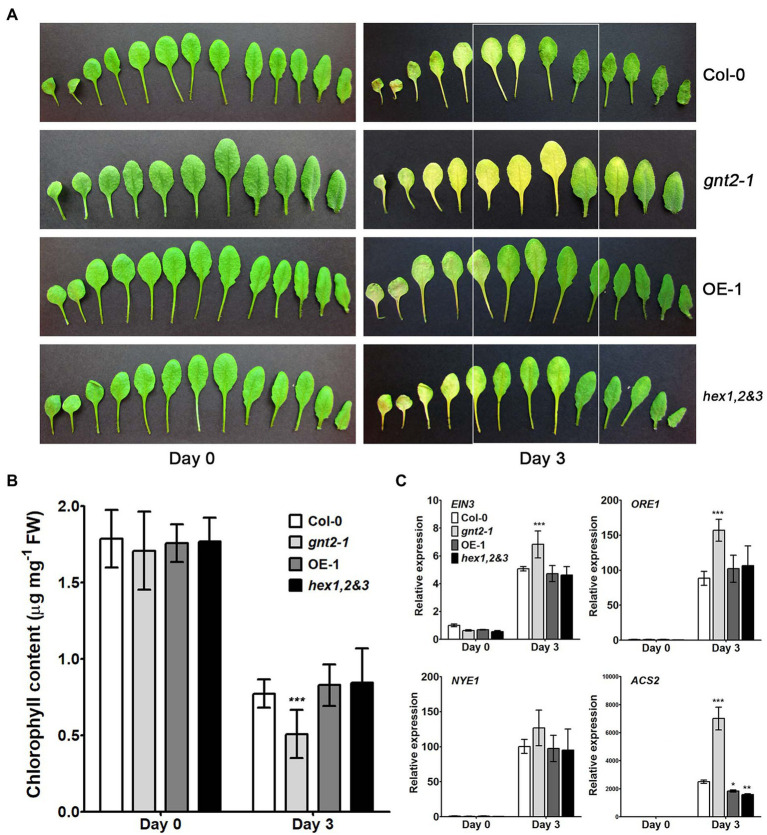
GnTII is involved in dark-induced leaf senescence. **(A)** Phenotypes of leaves from 4-week-old (0-day) Col-0, *gnt2-1*, OE-1, and *hex1,2&3* plants. Senescence symptoms of detached leaves from the Col-0, *gnt2-1*, OE-1, and *hex1,2&3* plants after 3day of dark treatment. The leaves are arranged from oldest (left) to youngest (right). **(B)** Chlorophyll contents of Col-0, *gnt2-1*, OE-1, and *hex1,2&3* plants. Chlorophyll content was determined in leaves from 4-week-old (0-day) plants and leaves following a 3-day dark treatment. **(C)** Relative expression of the senescence-related genes *EIN3*, *ORE1*, *NYE1*, and *ACS2* in leaves from the Col-0, *gnt2-1*, OE-1, and *hex1,2&3* plants analyzed by qPCR. Total RNA was extracted from 4-week-old plants before and after 3day of dark treatment. The experiment was performed independently three times (three biological replicates) and normalized with respect to *β-TUBULIN* expression. Data represent mean±SEM of three independent experiments. Asterisks indicate statistically significant differences (^*^p<0.05, ^**^p<0.01, and ^***^p<0.001) as determined by two-way ANOVA.

Previous studies indicated that the β1,2-GlcNAc residues at the nonreducing termini of N-glycans are potentially involved fruit ripening and/or leaf senescence in plants (Ghosh et al., 2011; Fanata et al., 2013). Additionally, when the Col-0, *gnt2-1*, OE-1, and *hex1,2&3* plants were cultivated in soil, the *gnt2-1* mutant, but not the OE-1 or *hex1,2&3* plants, displayed an early flowering phenotype with reduced rosette leaf number and flowering time compared with Col-0 plants under long-day (LD) condition (Supplementary Figure S1B). Thus, we wondered if the absence of the 6-arm β1,2-GlcNAc residue of N-glycan is related to the onset of dark-induced senescence in *gnt2-1* plants. To address this question, we used a dark-induced senescence assay in which we exposed detached rosette leaves from 4-week-old Col-0, *gnt2-1*, OE-1, and *hex1,2&3* plants to darkness for 3day. After 3day in the dark, the younger leaves of the *gnt2-1* plants lost more chlorophyll than those of Col-0 plants. However, chlorophyll loss in younger leaves of OE-1 or *hex1,2&3* plants was comparable to that of Col-0 plants (Figures 4A,B). Ethylene-mediated chlorophyll degradation is related to the onset of senescence in plant leaves (Hortensteiner, 2006; Song et al., 2014; Qiu et al., 2015). To further verify the initiation of accelerated dark-induced senescence in *gnt2-1*, we used qPCR to examine the expression of several senescence-associated genes, such as *EIN3*, *ORE1*, *NYE1*, and *ACS2*, before and after dark treatment. The expression of senescence-associated genes was higher in *gnt2-1* plants than in Col-0 plants, but not in OE-1 or *hex1,2&3* plants (Figure 4C). These results are consistent with previous findings of increased shelf life of tomato (*Solanum lycopersicum*) fruits with RNAi-suppressed *β-D-N-acetylhexosaminidase* genes and early onset of dark-induced senescence in the rice *gnt1* mutant (Meli et al., 2010; Fanata et al., 2013). As a result, structural diversity of N-glycans is important for normal plant growth and longevity.

**Figure 5:**
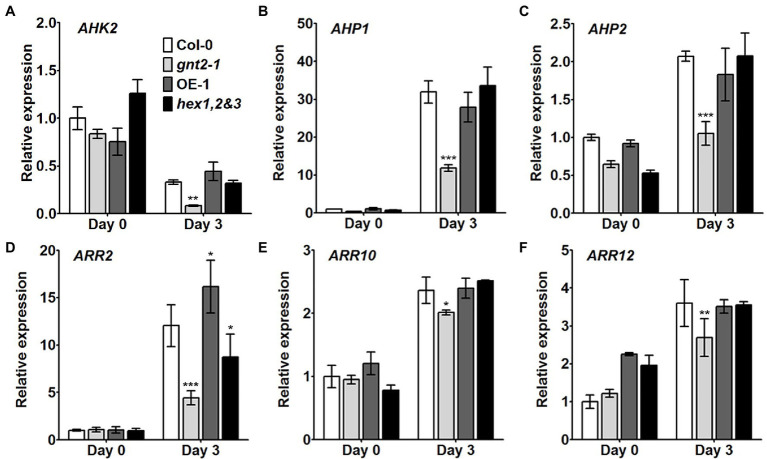
Cytokinin signaling is impaired in the *gnt2-1* mutant. **(A–F)** Relative expression of the cytokinin-related genes *AHK2* **(A)**, *AHP1* **(B)**, *AHP2* **(C)**, *ARR2* **(D)**, *ARR10* **(E)**, and *ARR12* **(F)** in leaves from Col-0, *gnt2-1*, OE-1, and *hex1,2&3* plants analyzed by qPCR. Total RNA was extracted from 4-week-old plants before and after 3day of dark treatment. The experiment was performed independently three times (three biological replicates) and normalized with respect to *β-TUBULIN* expression. Data represent mean±SEM of three independent experiments. Asterisks indicate statistically significant differences (^*^p<0.05, ^**^p<0.01, and ^***^p<0.001) as determined by two-way ANOVA.

Cytokinins have been shown to inhibit chlorophyll breakdown in tissues, which delays leaf senescence (Gan and Amasino, 1995; Kim et al., 2006). We used qPCR to examine the expression of cytokinin-related genes, such as *AHK2*, *AHP1*, *AHP2*, *ARR2*, *ARR10*, and *ARR12,* in the detached rosette leaves of 4-week-old Col-0, *gnt2-1*, OE-1, and *hex1,2&3* plants before and after a 3-day dark treatment to see if they were involved in the early onset of dark-induced senescence in *gnt2-1* plants (Figure 5). In the senescent leaves of plants of all four genotypes after dark treatment, the expression levels of *AHP1*, *AHP2*, *ARR2*, *ARR10*, and *ARR12* were increased (Figures 5B–F), while the expression of *AHK2* was decreased (Figure 5A). Cytokinin-related gene expression was significantly lower in *gnt2-1* plants than in Col-0 plants after dark treatment (Figure 5). However, except for *ARR2*, the expression levels of the genes in the OE-1 and *hex1,2&3* plants were comparable to those in the Col-0 plants.

### The *gnt2-1* Plants Are Less Sensitivity to Benzylaminopurine Treatment Than Col-0 Plants

**Figure 6:**
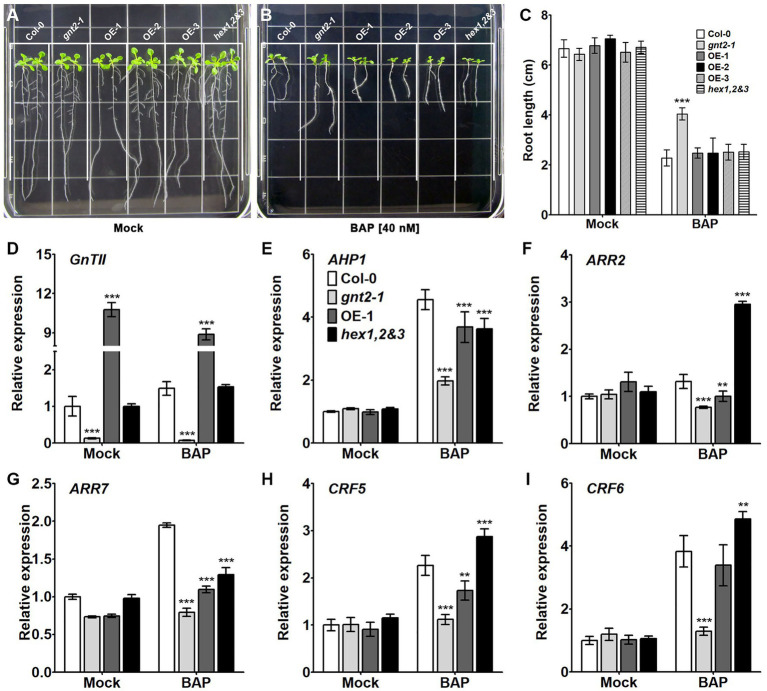
The *gnt2-1* mutant exhibits partial cytokinin insensitivity. **(A,B)** Representative photographs of Arabidopsis seedlings grown vertically on tissue culture plates containing solidified MS medium **(A)** or MS medium supplemented with 40nm BAP **(B)**, taken 12day after plating. The grid on the square plate is 18×18mm. **(C)** Relative root length of Col-0, *gnt2-1*, OE-1, OE-2, OE-3, and *hex1,2&3* plants grown in the absence or presence of BAP. **(D–I)**. Expression of cytokinin-related genes in Col-0, *gnt2-1*, OE-1, and *hex1,2&3* plants. The relative expression of mRNAs encoding the cytokinin-related genes was analyzed by qPCR. The experiment was performed independently three times (three biological replicates) and normalized with respect to *β-TUBULIN* expression. Data represent mean±SEM of three independent experiments. Asterisks indicate statistically significant differences (^*^p<0.05, ^**^p<0.01, and ^***^p<0.001) as determined by two-way ANOVA.

We wondered if cytokinin responsiveness was altered in the *gnt2-1* mutant since altered cytokinin signaling is linked to the early onset of dark-induced senescence. To address this question, Col-0, *gnt2-1*, OE-1, OE-2, OE-3, and *hex1,2&3* plants were grown for 12day on either standard MS medium or MS medium containing 40nm benzylaminopurine (BAP), a synthetic cytokinin that induces plant growth and development responses (D’Aloia et al., 2011). The extent to which this exogenous cytokinin inhibited primary root growth was significantly less in *gnt2-1* plants than in Col-0, OE-1, OE-2, OE-3, or *hex1,2&3* plants (Figures 6A–C), suggesting that the reduced cytokinin responsiveness is responsible for altered cytokinin signaling and an earlier onset of dark-induced leaf senescence in *gnt2-1*. The Arabidopsis histidine protein kinase CKI1 has been shown to localize to the plasma membrane, and treatment of Arabidopsis protoplasts with an N-glycosylation inhibitor, TM, fully abolished CKI1-GFP expression (Hwang and Sheen, 2001). Thus, the changes in the structural diversity of N-glycans may have impact on the conformation, localization, and/or function of the histidine protein kinases, possibly resulting in decreased cytokinin responsiveness in *gnt2-1*.

To determine if the decreased cytokinin responsiveness in *gnt2-1* results in altered cytokinin signaling, we used qPCR to evaluate the expression of cytokinin-regulated genes in Col-0, *gnt2-1*, OE-1, and hex1,2&3 plants grown on standard MS medium or MS medium containing 40nm BAP for 12day. In comparison with mock-treated plants, *AHP1*, *ARR7*, *CRF5*, and *CRF6* expression levels appeared to be higher in the presence of BAP in all four genotypes (Figures 6E,G–I). *ARR2* expression was increased only in the presence of BAP in the *hex1,2&3* plants (Figure 6F). However, the degree to which *AHP1*, *ARR7*, *CRF5*, and *CRF6* gene expression changed in *gnt2-1* in the presence or absence of BAP was less than in Col-0, OE-1, or *hex1,2&3* plants. Thus, the decreased cytokinin responsiveness results in altered cytokinin signaling in *gnt2-1*, which may be a consequence of changes in the structural diversity of N-glycans caused by the *GnTII* loss-of-function mutation.

### GnTII Loss-of-Function Mutation Impairs Auxin Transport

**Figure 7:**
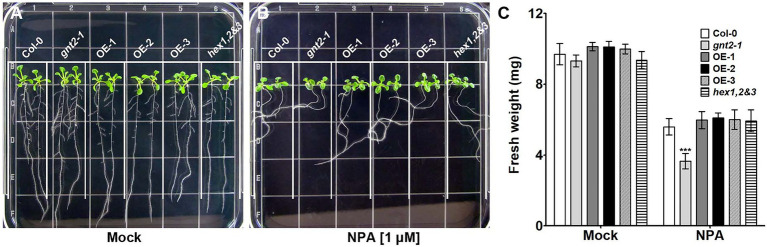
The *gnt2-1* mutant exhibits increased sensitivity to inhibition by NPA. **(A,B)** Representative photographs of Arabidopsis seedlings grown vertically on tissue culture plates containing solidified MS medium **(A)** or MS medium supplemented with 1μm NPA **(B)**, taken 12day after plating. The grid on the square plate is 18×18mm. **(C)** Fresh weight of Col-0, *gnt2-1*, OE-1, OE-2, OE-3, and *hex1,2&3* plants grown in the absence or presence of NPA. Asterisks indicate statistically significant differences (^*^p<0.05, ^**^p<0.01, and ^***^p<0.001) as determined by two-way ANOVA.

**Figure 8:**
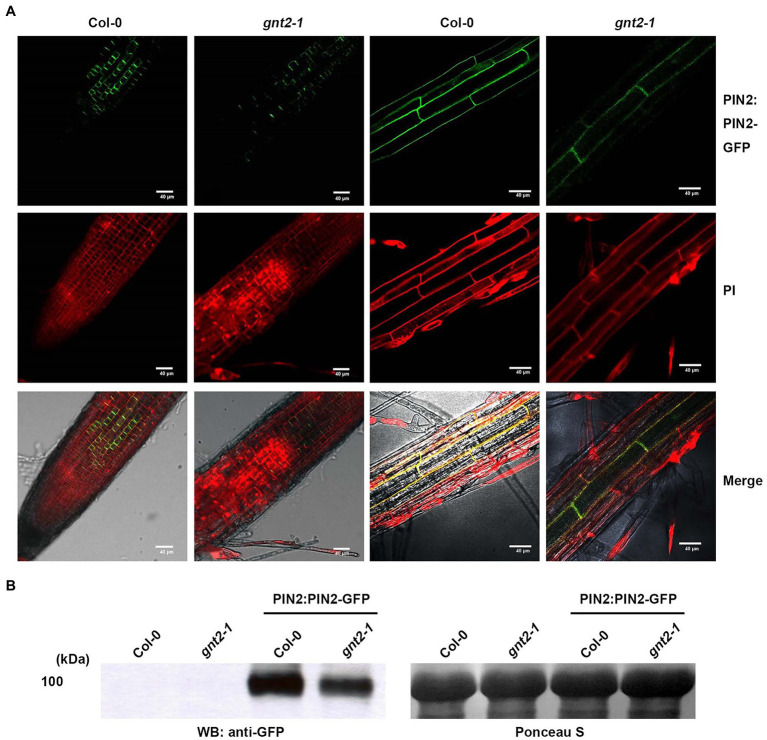
The stability and subcellular localization of PIN2-GFP are changed in *gnt2-1* under stress conditions. **(A,B)** Arabidopsis seedlings grown on tissue culture plates containing MS medium supplemented with 60mm NaCl for 7day were used. **(A)** The expression of PIN2:PIN2-GFP in Col-0 and *gnt2-1* background. The PIN2:PIN2–GFP localization in the root apical tissues of Col-0 and *gnt2-1* was visualized under a confocal laser scanning microscope (FV1000; Olympus). Scale bar=40μm. **(B)** Immunoblot analysis of PIN:PIN2-GFP in Col-0 and *gnt2-1* mutant background. The blots were probed with a monoclonal GFP antibody. Ponceau S staining shows equal protein loading.

The decreased cytokinin responsiveness of *gnt2-1*, which could be explained by changes in the structural diversity of N-glycans, indicated that the mutant plants could also have altered auxin transport. To determine whether *gnt2-1* exhibited an altered auxin response, Col-0, *gnt2-1*, OE-1, and *hex1,2&3* plants were grown for 12day on standard MS medium or on MS medium containing 1μm N-1-naphthylphthalamic acid (NPA), a chemical inhibitor that blocks polar auxin transport (PAT) by interfering with the cycling of auxin transporters, like PIN1 (Geldner et al., 2001; Abas et al., 2021). Roots were allowed to expand against gravity along the agar surface by orienting plates vertically (Figure 7). Positive gravitropism was more impaired in *gnt2-1* seedlings than in Col-0 seedlings following NPA treatment. However, positive gravitropism of OE-1 or *hex1,2&3* seedlings was comparable to that of Col-0 seedlings (Figures 7A,B). Additionally, NPA treatment substantially decreased total fresh weight in *gnt2-1* seedlings compared to Col-0 seedlings. The total fresh weights of OE-1, OE-2, OE-3, and *hex1,2,&3* seedlings, on the other hand, were similar to those of Col-0 seedlings (Figure 7C). Under salt stress, the expression of PIN2–green fluorescent protein (GFP) signals in the root apical meristem of Col-0 and *gnt2-1* seedlings was investigated to see what caused *gnt2-1* seedlings to be more sensitive to NPA. The basal membrane in the root apical tissues of Col-0 seedlings exhibited a polarized distribution of PIN2–GFP (Figure 8A). While basal PIN2–GFP localization was still observed in the root apical tissues of *gnt2-1* seedlings, PIN2–GFP signals were diffusely distributed across the plasma membrane (Figure 8A). Additionally, the intensity of PIN2–GFP signals was weaker in the root apical meristem of *gnt2-1* than in Col-0 (Figures 8A,B). These results indicate that *GnTII* loss-of-function mutation may affect the structural integrity of auxin transport carriers, such as PINs. Thus, changing the structural diversity of N-glycan in *gnt2-1* may affect a number of proteins involved in development or stress response.

## Discussion

Given the complexity of the natural environment in which plants grow and develop under abiotic and biotic challenges, the involvement of complex N-glycans in plant growth and development under natural field conditions may be critical. The analysis of Arabidopsis GnTII loss-of-function mutants (*gnt2-1* and *gnt2-2*) reveals that the addition of the 6-arm nonreducing β1,2-GlcNAc residue and the synthesis of the minor N-glycan structure are required for Arabidopsis growth and development under stress conditions (Figures 1, 2, and Supplementary Figure S7). In response to salt stress and ER stress, the *gnt2-1* and *gnt2-2* mutants had significantly lower root and shoot development rates than Col-0 (Figures 1, 2, and Supplementary Figure S7), as well as decreased sensitivity to cytokinin and NPA (Figures 6–8, Supplementary Figure S7). Thus, our findings are consistent with previous studies that found a link between N-glycan processing in the Golgi apparatus and plant development, both of which are coordinated by phytohormone signaling and homeostasis (Liebminger et al., 2009; Fanata et al., 2013; Harmoko et al., 2016; Liu et al., 2018).

ER stress is characterized by the accumulation of unfolded proteins in the ER lumen as a result of stress-induced disruption of ER function. When eukaryotic cells are exposed to ER stress, they activate a signaling pathway known as the UPR (Howell, 2013). In response to ER stress, plant cells have been found to activate at least two branches of the UPR signaling pathways: one mediated by IRE1-bZIP60 and another by site-1/site-2 proteases - bZIP17/bZIP28 (Liu et al., 2007a,b; Nagashima et al., 2011). In Arabidopsis, salt stress activates a signaling cascade that includes the processing of bZIP17, its translocation to the nucleus, and the activation of stress-related genes (Liu et al., 2007b). Salt stress increases BIP3 transcription, but it has little effect on the transcription of other ER chaperone genes such CRT1, CRT2, or PDIL-1 (Henriquez-Valencia et al., 2015). Furthermore, overexpression of an ER small heat-shock protein enhances cellular salt tolerance while suppressing the expression of other ER molecular chaperones (Fu et al., 2016). These findings suggest that salt stress is associated with and regulates some components of the UPR signaling pathway in plants. Under our experimental conditions, the expression of UPR-related genes remained high in the *gnt2-1* mutant in response to ER stress but did not change significantly by salt stress (Figure 3). Additionally, the *gnt2-1* and *gnt2-2* mutants exhibited increased susceptibility to salt stress and ER stress (Figures 1B–I, Supplementary Figure S7), as well as activation of UPR-related genes in response to ER stress (Figure 3). Our results showed that despite increased expression of the UPR-related genes, protein folding stress was not alleviated during prolonged ER stress in *gnt2-1* (Figures 1, 3). These data suggest that increased transcription of UPR-related genes in plants does not always represent increased resistance to ER stress and that plants exposed to more severe stresses may have higher UPR-related gene expression.

The relative quantity of minor N-glycans in stressed and untreated plants is crucial for understanding how the minor N-glycans contribute to the stress response. To determine this, we examined the relative quantity of the minor N-glycans in Col-0 plants cultivated for 12days on MS media with or without 60mm NaCl. However, our experimental conditions did not support the existence of a difference in the relative abundance of the minor N-glycans between the two plants. TM-treated plants were excluded in the experiment due to the possibility that it could have a direct effect on the amount of N-glycans found in plants. However, additional research is required to identify how the minor N-glycan levels alter in response to stress and what role they play in stress responses.

Phytohormones like as ethylene and cytokinins have been implicated in the regulation of leaf senescence (Aharoni and Lieberman, 1979; Bleecker et al., 1988; Gan and Amasino, 1995; Kim et al., 2006). Although ethylene has long been thought to be the primary hormone regulating the onset of leaf senescence and fruit ripening, increased cytokinin production may postpone leaf senescence (Aharoni and Lieberman, 1979; Gan and Amasino, 1995; Mccabe et al., 2001; Kim et al., 2006). Our findings reveal that Arabidopsis requires the minor N-glycan to delay dark-induced leaf senescence (Figures 4A,B). We found that dark-induced leaf senescence in *gnt2-1* activates the expression of genes involved in the ethylene signaling pathway, including EIN3, ORE1, NYE1, and ACS2 (Figure 4C). Additionally, the results of this study demonstrate that expression of genes involved in cytokinin signal transduction is significantly lower in *gnt2-1* than in Col-0 following dark treatment (Figure 5). As a result of our findings, we hypothesize that the minor N-glycan is required for the proper expression of genes or proteins involved in cytokinin signal transduction. Cytokinin inhibits primary root elongation and lateral root formation when applied exogenously (Li et al., 2006). The results of this study demonstrate that the *gnt2-1* mutant is less sensitive to the effects of an exogenously applied cytokinin (BAP) on primary root growth than Col-0 (Figures 6A–C). CKI1 and AHK3 are localized to the plasma membrane in Arabidopsis, indicating that a cytokinin signal is perceived at the plasma membrane *via* cytokinin receptors including CKI1 and AHK3 (Hwang and Sheen, 2001; Caesar et al., 2011). Additionally, treatment with an N-glycosylation inhibitor, TM, completely abolishes CKI1-GFP expression in Arabidopsis, and transient expression of AHK3-GFP fusion proteins in tobacco demonstrated AHK3 N-glycosylation (Hwang and Sheen, 2001; Caesar et al., 2011). However, it has been reported that the cytokinin receptors AHK2, AHK3, and AHK4 localize to both the ER and plasma membranes (Caesar et al., 2011; Wulfetange et al., 2011). Thus, additional studies will be required to determine whether the phenotype of decreased cytokinin sensitivity caused by the absence of the minor complex N-glycan in *gnt2-1* is associated with cytokinin receptors or with other putative GnTII substrates, such as active cytokinin transporters.

Auxins are essential for plant development and the primary phytohormone involved in gravitropism (Arnon, 1949; Cooper et al., 2001). Auxin biosynthesized in the shoot apex is transported to the basal parts of plants through PAT, which is a major mechanism for transporting auxin in the vascular meristem (Goldsmith, 1977; Jones, 1998; Muday and DeLong, 2001). *Via in situ* visualization, PAT was found to be correlated with the asymmetric distribution of Arabidopsis auxin efflux carriers PIN1 and PIN2 in the plasma membrane (Galweiler et al., 1998; Muller et al., 1998). PIN2–GFP expression in the root apical meristem of *gnt2-*1 seedlings was more diffusely distributed throughout the plasma membrane than in Col-0 seedlings during salt stress. Additionally, the quantity of PIN2–GFP in the root apical meristem of *gnt2-1* was much less than that in Col-0 (Figures 8A,B). Altered N-glycan structure, particularly the lack of the minor N-glycan in *gnt2-1*, may affect protein properties, such as stability and subcellular localization. As a consequence, the altered structure, stability, and localization of auxin transporters can affect PAT in *gnt2-1*, which in turn can affect plant development under stress conditions.

Along with the *gnt2-1* mutant identified in the Col-0 background, another loss-of-function mutation (*gnt2-2*) in *GnTII* was identified in Wassilewskija (WS) background. The *gnt2-2* (Flag_394A11) mutant obtained from National Institute for Agricultural Research (INRA) was used to confirm that the observed phenotype in the *gnt2-1* mutant is related to the *GnTII* loss-of-function mutation. The phenotypic effects of the loss-of-function mutation (*gnt2-2*) in *GnTII* were investigated in the absence or presence of TM, NaCl, and NPA (Supplementary Figure S7). TM and NaCl treatment of *gnt2-2* resulted in a substantial inhibition of growth (Supplementary Figure S7). In addition, *gnt2-2* seedlings exhibited enhanced resistance to NPA-induced suppression of growth and development (Supplementary Figure S7). Similar phenotypic features seen in both mutants indicate that, regardless of their genetic backgrounds, the role of GnTII in transferring 6-arm GlcNAc residue to N-glycans is required for plant growth and development under stress conditions.

In mice, knockout studies have revealed that the phenotypes associated with altered N-glycan structure become increasingly mild as the altered N-glycan structure becomes less extensive and also as the changed N-glycan structure nearer the nonreducing end (Gottlieb et al., 1975; Stanley et al., 1975; Vischer and Hughes, 1981). The elimination of all complex N-glycans is fatal in organisms but not in cultured cells, implying that they are involved in development (Ioffe and Stanley, 1994; Metzler et al., 1994). N-glycan maturation is required for cytokinin-mediated development and cellulose synthesis in rice, indicating that N-glycan maturation is involved in the transport and/or function of membrane proteins associated to cytokinin and cellulose (Fanata et al., 2013). N-glycan containing a core α1,3-fucose residue is required for basipetal auxin transport and gravitropic response in rice (Harmoko et al., 2016). The absence of the core α1,3-fucose residue may have an effect on the localization and/or function of the auxin transporters in the *fuct-1* and *fuct-2* mutants (Harmoko et al., 2016). The mutation of a single conserved N-glycosylation site in the Arabidopsis receptor-like kinase elongation factor Tu receptor promotes protein synthesis in the ER and inhibits ligand-binding activity in the plasma membrane (Haweker et al., 2010). A nonglycosylated mutant of Arabidopsis vacuolar sorting receptor 1 (AtVSR1) has a weaker affinity for the cargo than the wild-type AtVSR1, reducing the efficacy of cargo transport from the Golgi and ultimately resulting in cargo secretion (Kim et al., 2010). Thus, N-glycosylation of AtVSR1 is important for its function in plants as a vacuolar sorting receptor. Disruption of the minor N-glycan synthesis pathway resulted in a variety of abnormalities in our study, including hypersensitivity to stress and hyposensitivity to phytohormones. The pleiotropic defects found in the GnTII loss-of-function mutants may be due to altered intracellular localization and/or structure of the diverse family of glycoproteins containing N-glycan acceptors for GnTII. However, additional research will be required to comprehend the entire complexity of the molecular pathways behind the GnTII loss-of-function mutant phenotypes.

## Experimental Procedures

### Plant Materials and Growth Conditions

A full-length cDNA encoding GnTII was cloned from the Arabidopsis leaf cDNA library by PCR using a pair of gene-specific primers (Supplementary Table S1). The full-length cDNA of *GnTII* was cloned into a *pCAMBIA* vector under the control of the CaMV 35S promoter. The plasmid *pCAMBIA::35Sp:GnTII* containing the *hpt* gene as a selection marker was transformed into *Agrobacterium tumefaciens* strain GV3101. The Agrobacterium strain with the *pCAMBIA::35Sp:GnTII* was subsequently used to transform *A. thaliana* ecotype Columbia (Col-0) by the floral dip method (Geldner et al., 2001). A total of 50 independent transgenic lines (T0) were selected by hygromycin-resistance screening. The copy number and homozygosity of each transgenic line were determined by their mortality on the selection plates with hygromycin (25mgl^−1^). A total of 19 independent homozygous transgenic lines (T3) were identified. The seeds from three representative GnTII-overexpressing lines (OE-1, OE-2, and OE-3) were selected for further functional analyses. Mutant seeds of *gnt2-1* (Salk_063549) and *gnt2-2* (Flag_394A11) were obtained from the Arabidopsis Biological Resource Center and from the INRA, respectively, and screened by PCR with *GnTII* and T-DNA-specific primer pairs (Supplementary Table S1). The *PIN2:PIN2-GFP* transgenic line was obtained from Dr. Inhwan Hwang at Pohang University of Science and Technology, Korea (Abas et al., 2021). The *PIN2:PIN2-GFP* transgenic line was crossed to the *gnt2-1* knockout mutants to obtain *gnt2-1* mutants expressing the *PIN2:PIN2-GFP*. Arabidopsis Col-0, *gnt2-1*, OE-1, OE-2, OE-3, and *hex1,2&3* plants were grown in a growth chamber at 22°C under long-day conditions (16-h/8-h light/dark photoperiod, 100–200μmolm^−2^ s^−1^ photon flux density, and 60–70% relative humidity) on 1×Murashige and Skoog (MS) medium (Duchefa), pH 5.8, supplemented with 3% (w/v) sucrose and 0.25% (w/v) gellan gum (PhytoTechnology Laboratories). All seeds were cold-treated at 4°C for 4day in darkness and then incubated at 22°C.

### Stress and Hormone Treatment

Arabidopsis seeds were placed in Petri dishes on either standard solid MS medium or solid MS medium supplemented with 20μgl^−1^ TM, 60mm NaCl, 40nm BAP, or 1μm NPA and were allowed to germinate and grow. To analyze *GnTII* expression following treatment with NaCl and TM, seedlings were transferred to sterilized 3mm paper and submerged in 5ml standard liquid MS medium or liquid MS medium containing 200mm NaCl or 5mgl^−1^ TM (Kang et al., 2008; Nagashima et al., 2011). To synchronize sample harvesting, seedlings were treated with NaCl or TM for the indicated periods of time just prior to being harvested on the 17th day after imbibition.

### Staining of Callose Deposition in Root

Five-day-old plants were treated for 4day with 130mm NaCl and with 50μgl^−1^ TM at 22°C, respectively. To detect callose deposition, seedlings were immersed in 100mm K_2_HPO_4_ and 0.005% decolorized aniline blue for 2h in a Falcon tube wrapped in aluminum foil for light protection (Kang et al., 2008). Callose deposition was documented using a FV1000 confocal laser scanning microscope (Olympus) equipped with a DAPI filter set. The optimal excitation wavelength for aniline blue was 370nm, and the emission maximum was 509nm.

### Immunoblot and Lectin Blot Analyses

Tissue was ground in liquid nitrogen, resuspended in phosphate-buffered saline buffer (pH 7.4, 137mm NaCl, 10mm phosphate, and 2.7mm KCl) and cleared by centrifugation (10min at 15000×*g*). The protein content was determined using a protein assay kit (Bio-Rad) and bovine serum albumin as a standard. Each protein (20μg) was mixed with SDS–polyacrylamide gel electrophoresis (PAGE) loading buffer, denatured at 95°C for 5min, and subjected to 10% SDS–PAGE under reducing conditions. Separated proteins were either stained with Coomassie Brilliant Blue R-250 or transferred to a nitrocellulose membrane (Hybond-ECL, Amersham). Blots were blocked in 5% (w/v) non-fat dry milk in Tris-buffered saline (TBS) buffer (pH 7.6, 20mm Tris–HCl, and 137mm NaCl) for 1h and incubated in a 1:10,000 dilution of rabbit anti-horseradish peroxidase- (Sigma), anti-α1,3-fucose-, anti-β1,2-xylose antibodies (Agrisera), and anti-GFP (Abcam) in TBS supplemented with 0.1% (v/v) Tween 20. Detection was performed after incubation in a 1:3,000 dilution of a horseradish peroxidase-conjugated goat anti-rabbit antibody (Bio-Rad) in TBS-Tween with Western Blotting Detection Reagents (ECL, Amersham). For lectin blot analysis, concanavalin A (ConA, Sigma) was used to detect N-glycans with terminal mannose residues, and *Griffonia simplicifolia* lectin (GSII, EY laboratories) was used to detect N-glycans with terminal GlcNAc residues.

### N-glycan Isolation and Analysis by Mass Spectrometry

N-glycan purification from protein was performed as previously described (Yoo et al., 2015). The sodiated samples (treated with 1mm of NaOH in 80% MeOH) were directly infused into a 30-μm fused silica emitter (New Objective). The relative quantitation of N-glycan was performed on the Q Exactive^™^ Plus Orbitrap Mass Spectrometer (Thermo Scientific) equipped with a Nanospray Flex Ion Source for direct infusion at a 0.5μlmin^−1^ flow rate. The full MS spectra were obtained for quantification with 30-s data acquisition time in the 600- to 2,000-Da mass range. N-linked glycan structures were assigned using GlycoMod platform^1^ (Cooper et al., 2001).

### Quantitative PCR

Total RNA was extracted from seedlings using a NucleoSpin RNA Plant Kit (Macherey-Nagel) following the manufacturer’s instructions. For each sample, 1μg of purified RNA was used for first-strand cDNA synthesis using a ReverTraAce-α Kit (Toyobo) according to the manufacturer’s instructions. The synthesized cDNA samples were diluted (1:50) with sterile diethylpyrocarbonate -treated water. Quantitative PCR (qPCR) was performed with a CFX96 real-time PCR system (Bio-Rad). qPCR was conducted in a 10μl reaction volume including 0.5μl of each primer (10 pm), 4μl of template cDNA, and 5μl of iQ^™^ SYBR Green Supermix (Bio-Rad). The thermal profile used was as follows: 1cycle of 50°C for 2min and 95°C for 5min, followed by 40cycles of 95°C for 10s and 60°C for 30s. Finally, a melting-curve analysis (1cycle) from 65°C to 95°C was carried out. Expression data shown are the means±SEM of three independent biological replicates and were normalized to *β-TUBULIN* expression. Primers used in this study are presented in Supporting Information Supplementary Table S1.

### Dark Induction of Senescence in Detached Leaves

Plants were grown for 2week on solid MS medium, and then transferred to soil and grown for a further 2week. All green leaves were detached and placed on plastic trays wrapped with a double layer of aluminum foil. Petri dishes were kept in continuous darkness for 3day in a growth chamber at 22°C (Oh et al., 1996).

### Measurement of Chlorophyll Content

Chlorophyll was extracted by grinding the excised leaf tissue in 80% acetone (1ml 10mg^−1^ of tissue) with a ground-glass homogenizer. The homogenate was centrifuged at 1,500*g* for 5min, and the absorbance of the supernatant solution was determined at 663 and 645nm. Total chlorophyll contents were calculated according to the following equation (Arnon, 1949): total chlorophyll (mgl^−1^)=8.02(*A*_663_−*A*_710_)+20.2(*A*_645_−*A*_710_).

### Subcellular Localization and Expression Analysis of PIN2-GFP

Expression of PIN2-GFP was analyzed in the cell division and elongation zone of Arabidopsis roots. Roots were dipped in 10ng/ml propidium iodide (PI, Sigma) in H_2_O for 5min in the dark and then removed excess staining by rinsing in water. Fluorescence imaging was performed using a model FV1000 confocal laser scanning microscope (Olympus) with excitation at 488 and 543nm and emission at 510–540nm for GFP and 587–625nm for PI.

## Data Availability Statement

The original contributions presented in the study are included in the article/Supplementary Material, further inquiries can be directed to the corresponding author.

## Author Contributions

KL conceived, designed, and coordinated the study and wrote the paper. JY, KK, BV, YL, SY, YK, and JH performed and analyzed the experiments shown in Figures S1–S8, and in Supplementary Figures S1–S7. YK and JH provided assistance in the study and interpretation of the data. TP, JK, and JL performed and analyzed the experiments shown in Supplementary Figures S3–S6. All authors reviewed the results and approved the final version of the manuscript.

## Funding

This work was supported by the Cooperative Research Program for Agriculture Science and Technology Development (project no. PJ016236) and by the National Research Foundation of Korea (NRF, 2021R1A2C1013516, 2019R1I1A1A01058736, 2019R1A6A 3A01090824, 2020R1A6A1A03044344, and 2021R1I1A3060501) grants funded by the Korean government (Rural Development Administration, the Ministry of Science and ICT, and Ministry of Education). BV and YL were supported by the BK4 program funded by the Ministry of Education of Korea.

## Conflict of Interest

The authors declare that the research was conducted in the absence of any commercial or financial relationships that could be construed as a potential conflict of interest.

## Publisher’s Note

All claims expressed in this article are solely those of the authors and do not necessarily represent those of their affiliated organizations, or those of the publisher, the editors and the reviewers. Any product that may be evaluated in this article, or claim that may be made by its manufacturer, is not guaranteed or endorsed by the publisher.

## Footnotes

^1^ http://web.expasy.org/glycomod/

## Supplementary Material

The Supplementary Material for this article can be found at: https://www.frontiersin.org/articles/10.3389/fpls.2021.761064/full#supplementary-material
