# Supplementary material for: N-acetylglucosaminyltransferase II Is Involved in Plant Growth and Development Under Stress Conditions
Source: Front Plant Sci. 2021 Nov 2;12:761064. doi: 10.3389/fpls.2021.761064 (PMC8596550; doi:10.3389/fpls.2021.761064)
Supplement: Supplementary file 1 [file Data_Sheet_1.docx]

Supplementary Material

***N*-acetylglucosaminyltransferase II is involved in plant growth and development under stress conditions**

Jae Yong Yoo^2†^, Ki Seong Ko^2†^, Bich Ngoc Vu^2,3^, Young Eun Lee^2,3^, Seok Han Yoon^2,3^, Thao Thi Pham^5^, Ji-Yeon Kim^5^, Jae-Min Lim^5^, Yang Jae Kang^1,2,4^, Jong Chan Hong^1,2,3^, and Kyun Oh Lee^1,2,3^*

**Contents**

**Supplementary Figure 1. Gene expression and phenotypic traits of Col-0, *gnt2-1*, GnTII-overexpression lines, and *hex1,2&3*.**

**Supplementary Figure 2. Antibodies recognizing α1,3-fucose and/or β1,2-xylose residues exhibit different interaction patterns against proteins from Col-0, *gnt2-1*, OE-1, and *hex1,2&3* plants.**

**Supplementary Figure 3. Orbitrap-based MS analysis of the PNGase-A‐released permethylated N‐glycans from Col-0 plants.**

**Supplementary Figure 4. Orbitrap-based MS analysis of the PNGase-A‐released permethylated N‐glycans from *gnt2-1* plants.**

**Supplementary Figure 5. Orbitrap-based MS analysis of the PNGase-A‐released permethylated N‐glycans from OE-1 plants.**

**Supplementary Figure 6. Orbitrap-based MS analysis of the PNGase-A‐released permethylated N‐glycans from *hex1,2&3* plants.**

**Supplementary Figure 7. The *gnt2-2* mutant exhibits altered responses to TM, NaCl and NPA**

**Supplementary Table 1. Primers used in this study**

**Figure S1**

**
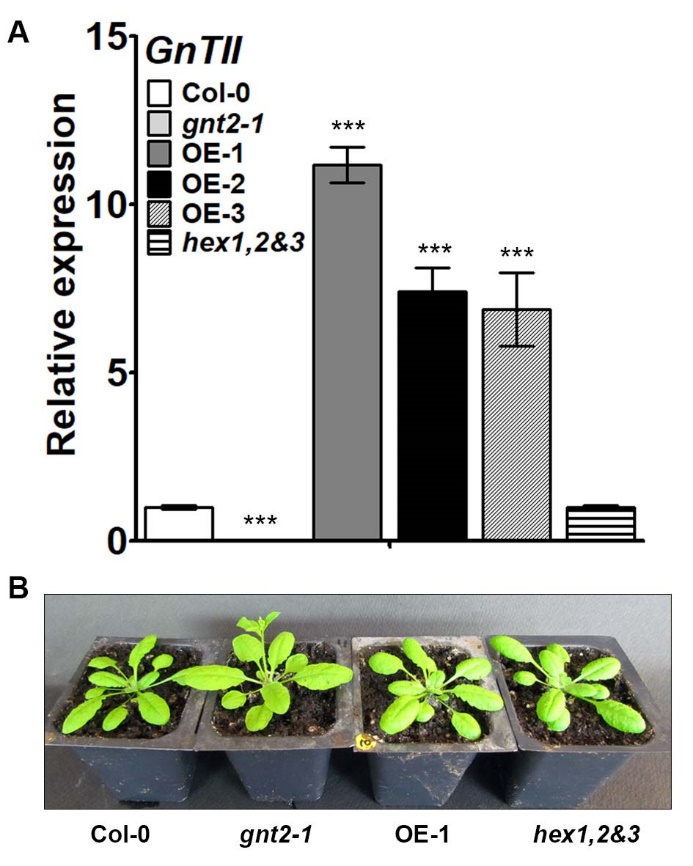
**

**Supplementary Figure 1. Gene expression and phenotypic traits of Col-0, *gnt2-1*, GnTII-overexpression lines, and *hex1,2&3*.** (A) Quantitative real-time PCR (qRT-PCR) analysis of *GnTII* in Col-0, *gnt2-1*, OE-1, OE-2, OE-3, and *hex1,2&3* plants. Total RNA was isolated from 2 weeks old Arabidopsis plants. The experiment was performed independently three times (three biological replicates) and normalized with respect to *β-TUBULIN* expression. Asterisks indicate statistically significant differences (*p < 0.05, **p < 0.01, ***p < 0.001) as determined by two-way analysis of variance (ANOVA). (B) 24-day-old Col-0, *gnt2-1*, OE-1, and *hex1,2&3* plants grown in soil under normal conditions.


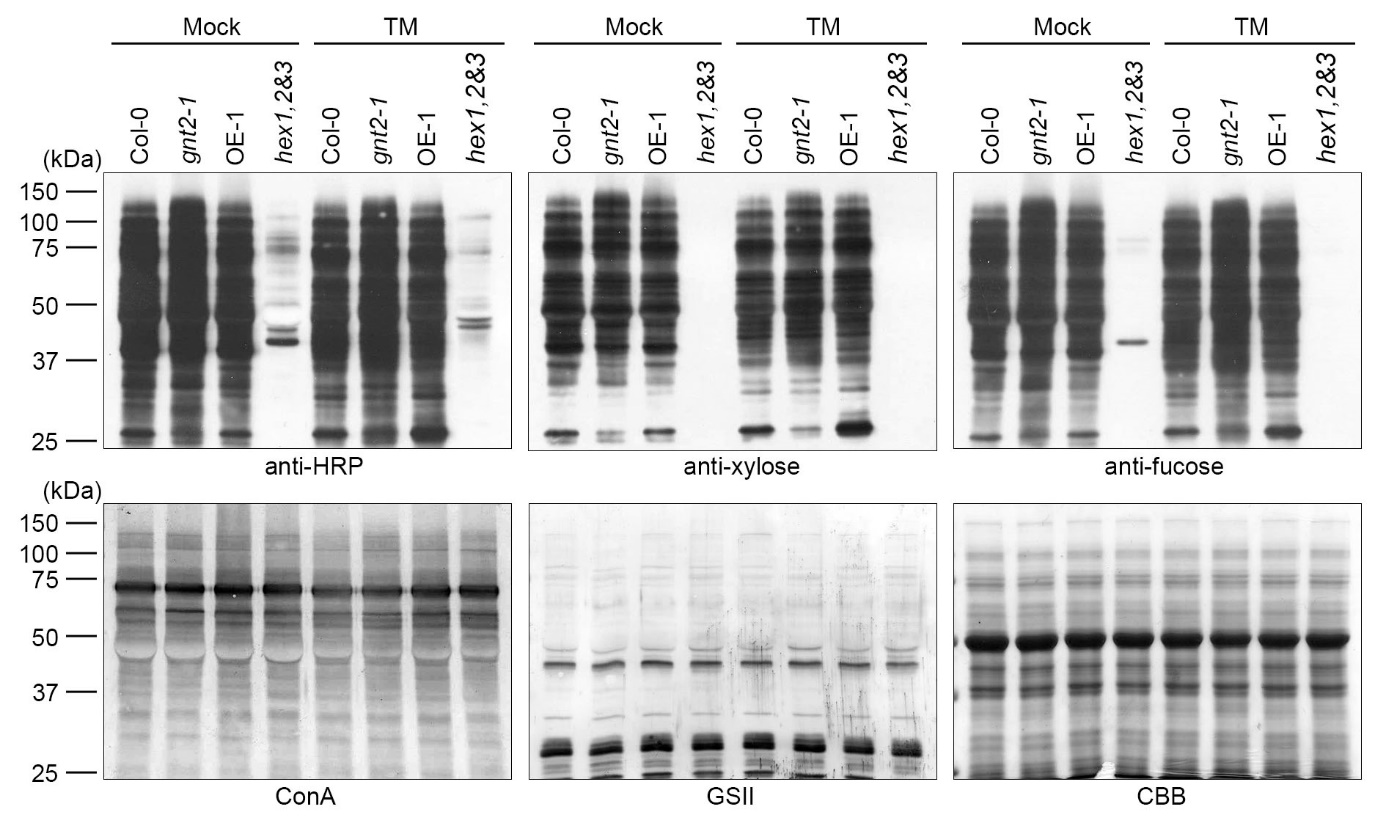


**Supplementary Figure 2. Antibodies recognizing α1,3-fucose and/or β1,2-xylose residues exhibit different interaction patterns against proteins from Col-0, *gnt2-1*, OE-1, and *hex1,2&3* plants.** Immunoblot and lectin blot analyses of total leaf protein from Col-0, *gnt2-1*, OE-1, and *hex1,2&3* plants. Total proteins extracted from 2-week-old seedlings were subjected to immunoblot and lectin blot analyses. The immunoblots were probed with anti-HRP, anti-xylose, and anti-fucose antibodies, and lectin blots were probed with ConA and GSII. Coomassie Brilliant Blue (CBB) staining was used to show equal loading of the proteins.


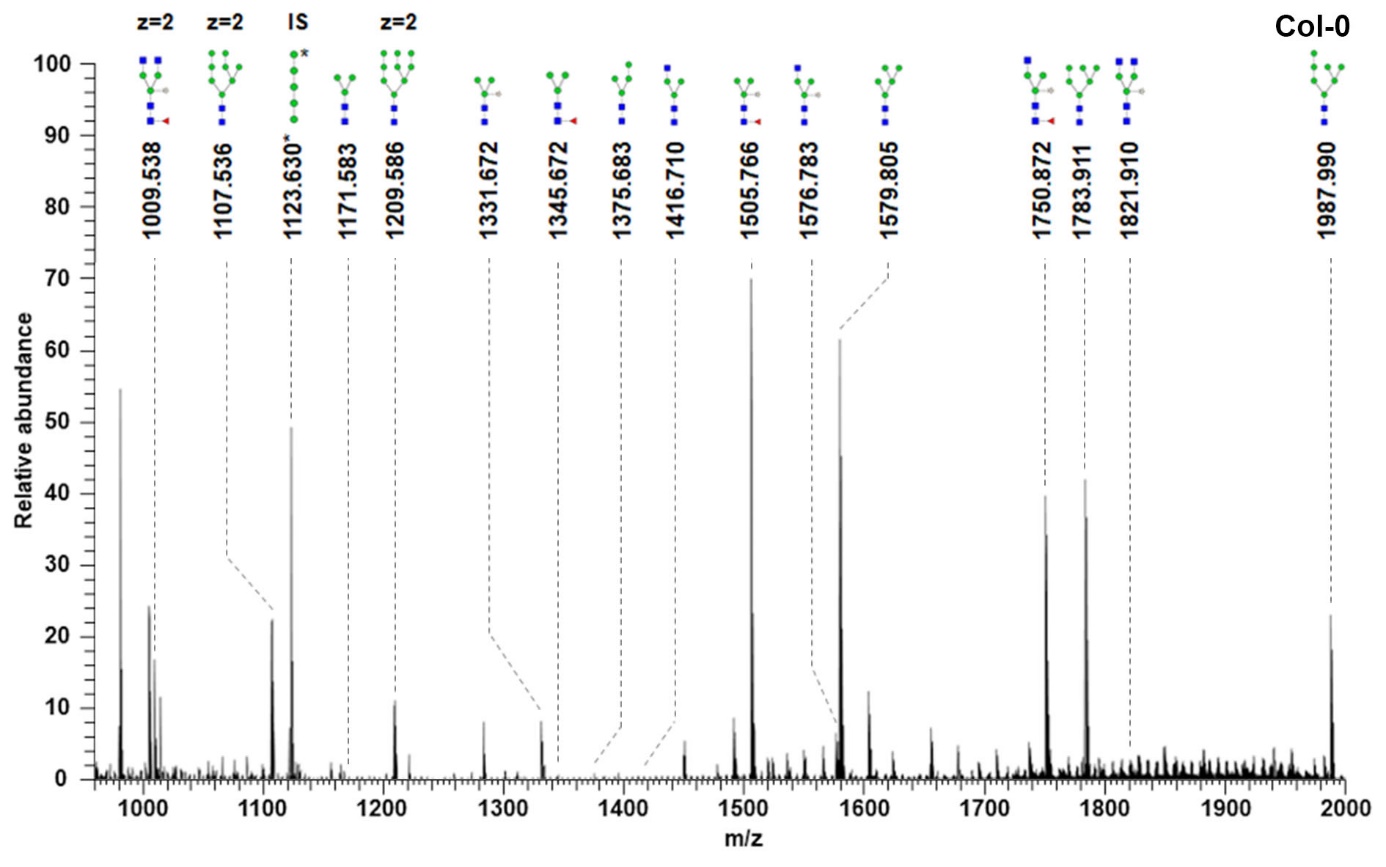


**Supplementary Figure 3. Orbitrap-based MS analysis of the PNGase-A‐released permethylated N‐glycans from Col-0 plants.** The proposed N‐glycan structures of the peaks are shown, along with their corresponding *m*/*z* value. Internal standards (IS) are indicated by asterisks. Blue squares, *N*‐acetylglucosamine; green circles, mannose; gray stars, xylose; red triangles, fucose.


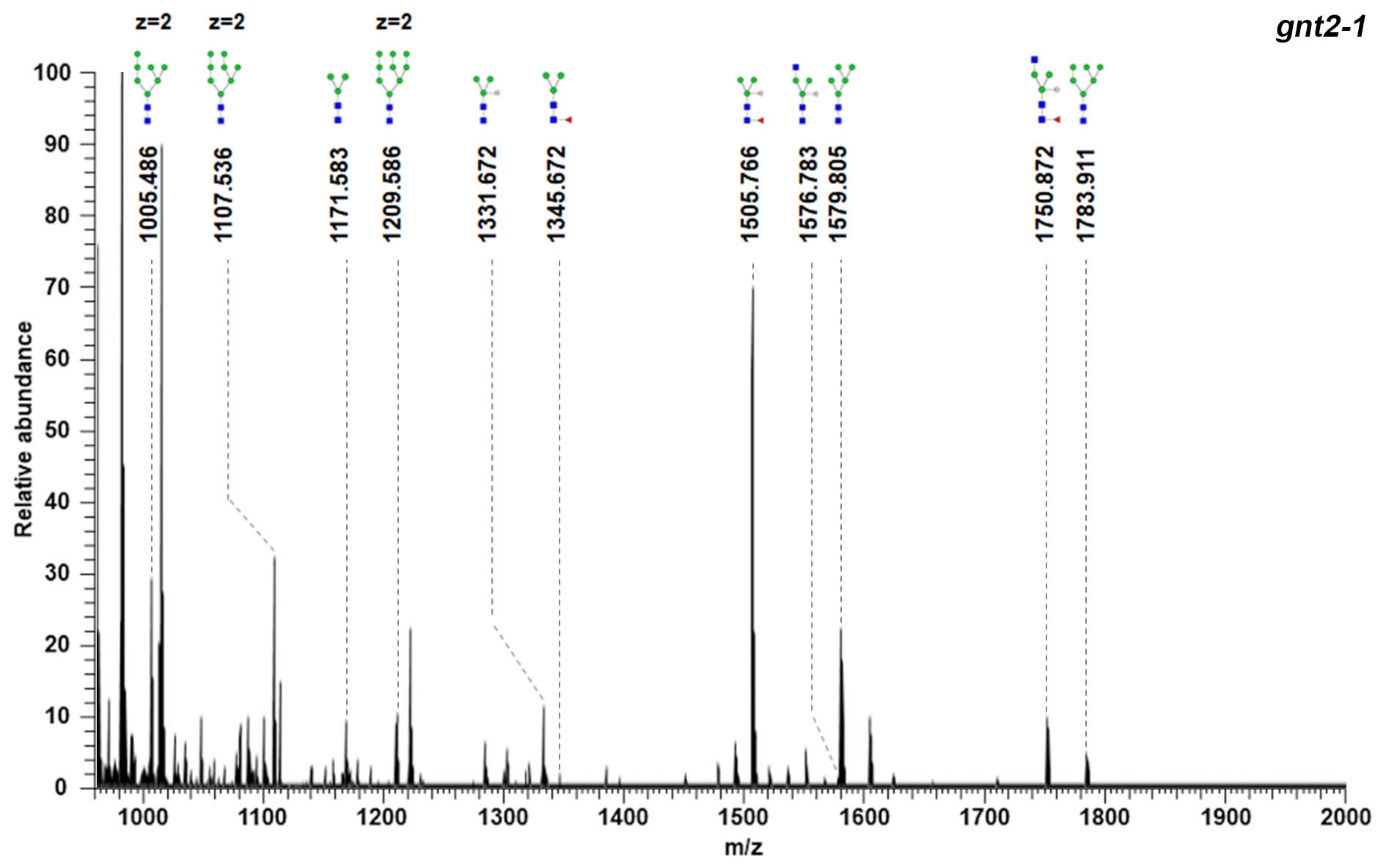


**Supplementary Figure 4. Orbitrap-based MS analysis of the PNGase-A‐released permethylated N‐glycans from *gnt2-1* plants.** The proposed N‐glycan structures of the peaks are shown, along with their corresponding *m*/*z* value. Internal standards (IS) are indicated by asterisks. Blue squares, *N*‐acetylglucosamine; green circles, mannose; gray stars, xylose; red triangles, fucose.


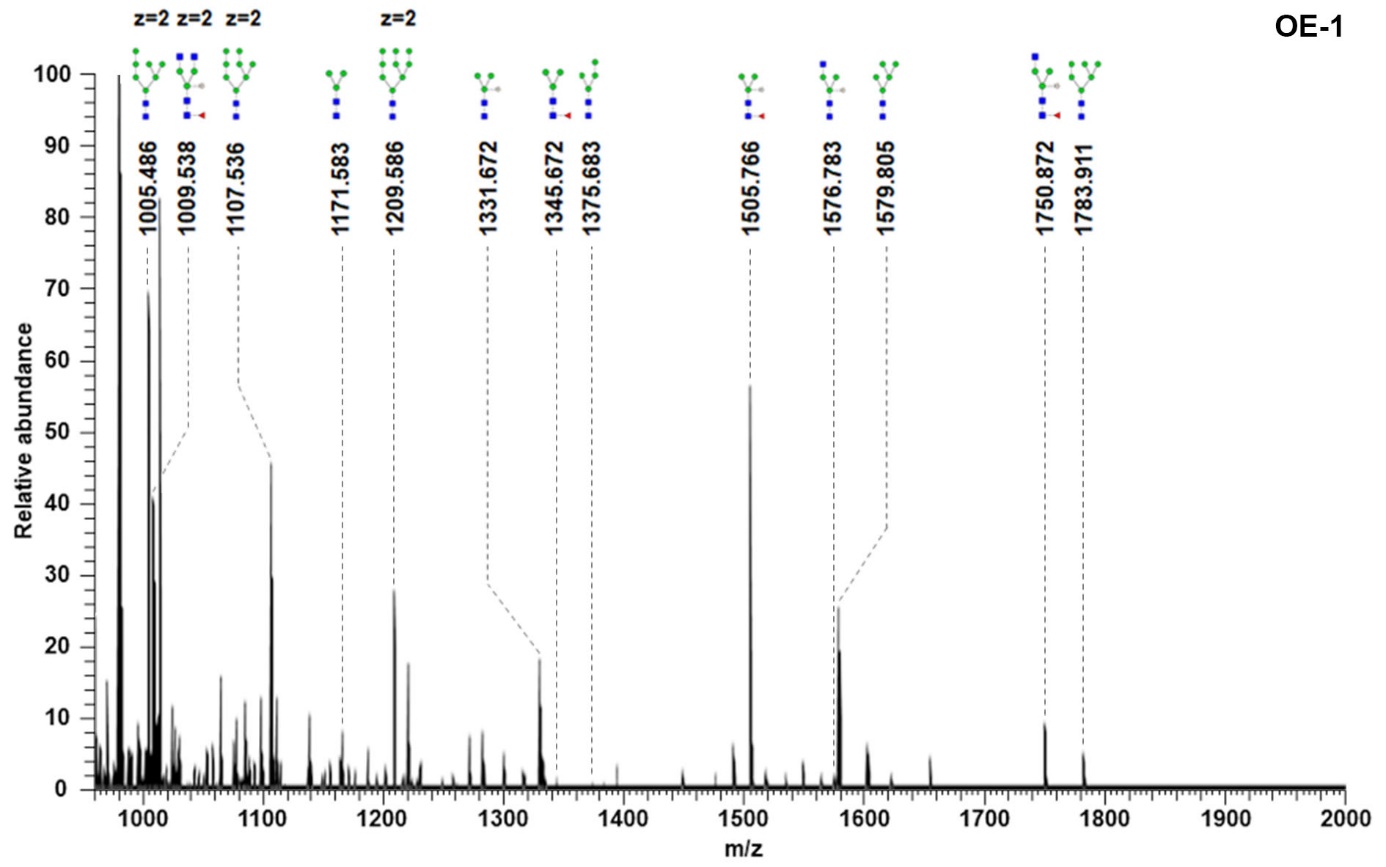


**Supplementary Figure 5. Orbitrap-based MS analysis of the PNGase-A‐released permethylated N‐glycans from OE-1 plants.** The proposed N‐glycan structures of the peaks are shown, along with their corresponding *m*/*z* value. Internal standards (IS) are indicated by asterisks. Blue squares, *N*‐acetylglucosamine; green circles, mannose; gray stars, xylose; red triangles, fucose.


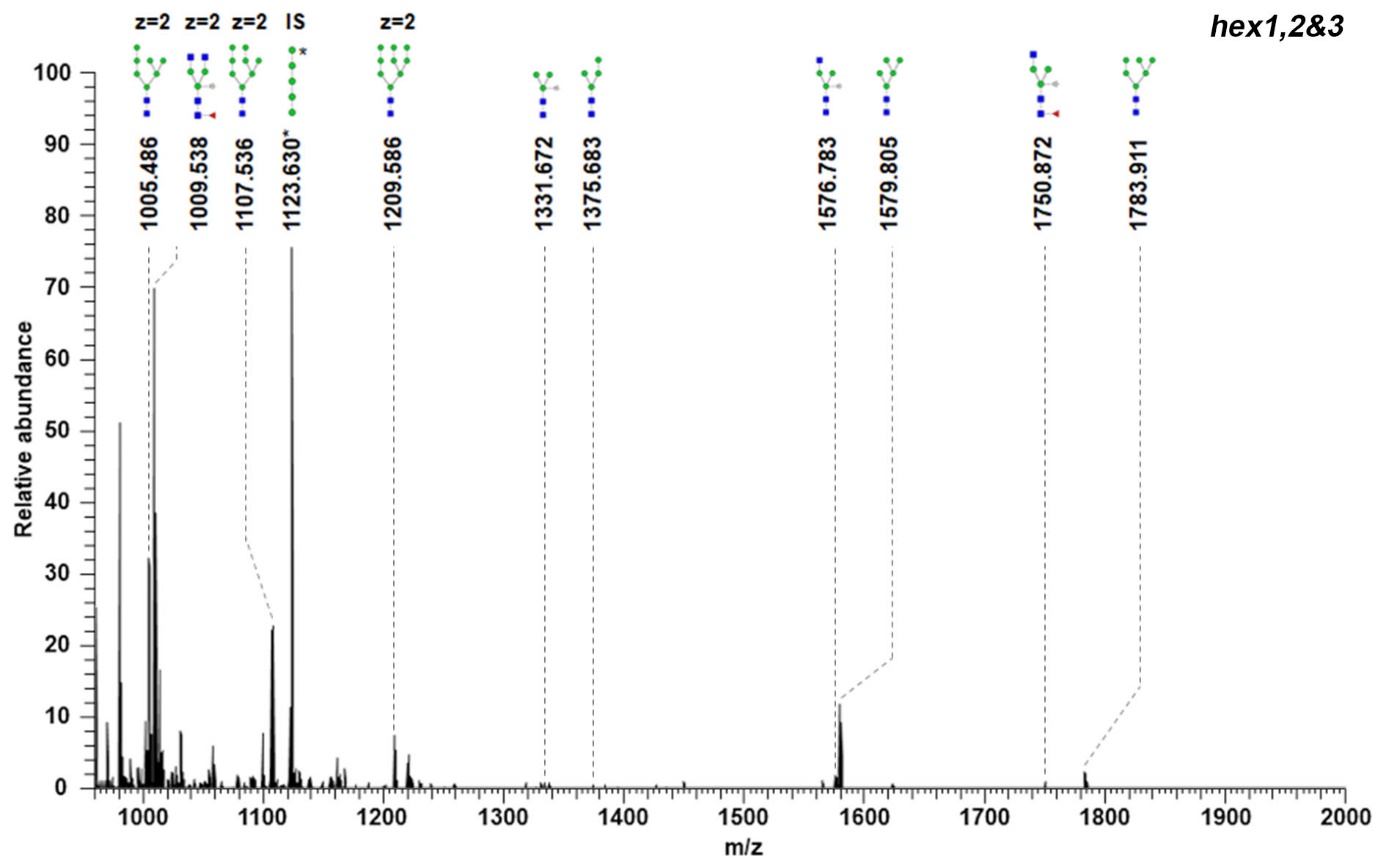


**Supplementary Figure 6. Orbitrap-based MS analysis of the PNGase-A‐released permethylated N‐glycans from *hex1,2&3* plants.** The proposed N‐glycan structures of the peaks are shown, along with their corresponding *m*/*z* value. Internal standards (IS) are indicated by asterisks. Blue squares, *N*‐acetylglucosamine; green circles, mannose; gray stars, xylose; red triangles, fucose.


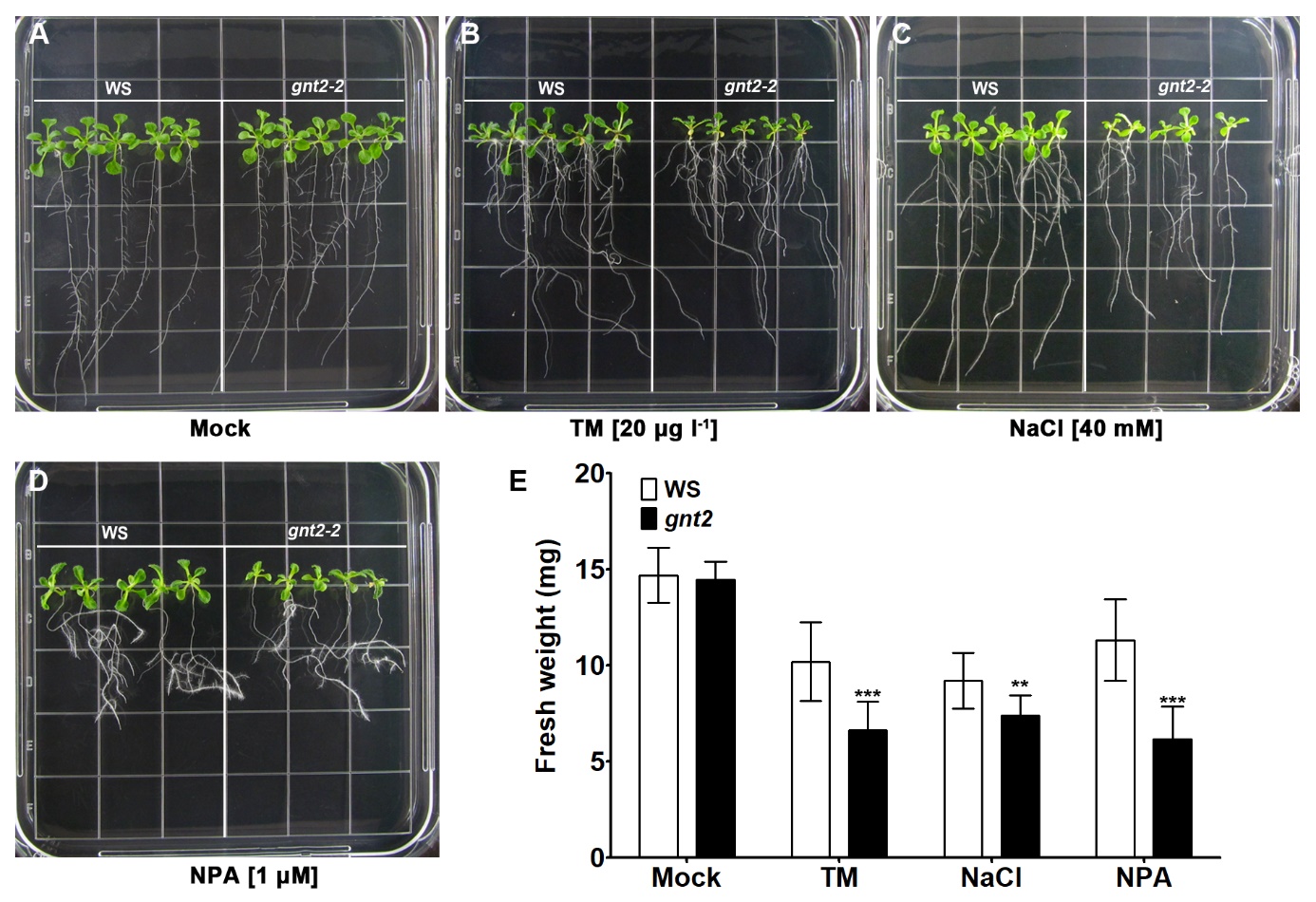
**Supplementary Figure 7. The *gnt2-2* mutant exhibits altered responses to TM, NaCl and NPA.** (A-D). Representative photographs of Arabidopsis seedlings grown vertically on tissue culture plates containing solidified MS medium (A) or MS medium supplemented with 20 μg l^−1^ TM (B) or 40 mM NaCl (C), 1 μM NPA (D), taken 2 weeks after plating. The grid on the square plate is 18 × 18 mm. (E) Fresh weight of WS, *gnt2-2* plants grown in the absence or presence of TM, NaCl, NPA. Asterisks indicate statistically significant differences (*p < 0.05, **p < 0.01, ***p < 0.001) as determined by two-way analysis of variance (ANOVA).

**Supplementary Table 1. Primers used in this study**

| Primer name | Primer sequence (5´ to 3´) | Associated gene | Use |
| --- | --- | --- | --- |
| GnTII F | 5'-ACAAGGGTGATGAGGCAAAG-3' | *GnTII* | qPCR |
| GnTII R | 5'-TCCAACCCATCCCATACAGT-3' | *GnTII* | qPCR |
| BiP3 F | 5'-GAGAAGAAACACAAAATATTCTACTG-3' | *BIP3* | qPCR |
| BiP3 R | 5'-CCTCGGAATAATGTTTGTCATC-3' | *BIP3* | qPCR |
| CNX1 F | 5'-GCTTTAGTTTCGTTCCAGA-3' | *CNX* | qPCR |
| CNX1 R | 5'-TGCTTCCATACACCTTCGTA-3' | *CNX* | qPCR |
| CRT1b F | 5'-GCAAATCAAGAACCCCAAC-3' | *CRT* | qPCR |
| CRT1b R | 5'-CGAGTCCAACATATTTCAGC-3' | *CRT* | qPCR |
| PDIL2-2 F | 5'-CCAATTTGCTGTCTGTTGTTT-3' | *PDIL* | qPCR |
| PDIL2-2 R | 5'-AACGACTCCATTTGAATTAAGAAC-3' | *PDIL* | qPCR |
| bZIP60 F | 5'-GAAGGAGACGATGATGCTGTGGCT-3' | *bZIP60* | qPCR |
| bZIP60 UR | 5'-GCAGGGATTCCAACAAGAGCACAG-3' | *bZIP60* | qPCR |
| bZIP60 SR | 5'-AGCAGGGAACCCAACAGCAGACT-3' | *bZIP60* | qPCR |
| bZIP28 F | 5'-TGATATTGCTCCTGGTGCTA-3' | *bZIP28* | qPCR |
| bZIP28 R | 5'-TTCTCTTGCCGTGGGTAG-3' | *bZIP28* | qPCR |
| bZIP17 F | 5'-CGTGAAGGTGTTGCAGGTC-3' | *bZIP17* | qPCR |
| bZIP17 R | 5'-ACGTTGGTAGCTGCAGGAAT-3' | *bZIP17* | qPCR |
| *TUBULIN* F | 5'-ATCGATTCCGTTCTCGATGT-3' | *β-TUBULIN* | qPCR |
| *TUBULIN* R | 5'-ATCCAGTTCCTCCTCCCAAC-3' | *β-TUBULIN* | qPCR |
| ARR2 F | 5'- ACACGTTGGTTTAGAGATGG -3' | *ARR2* | qPCR |
| ARR2 R | 5'- TACGTACCGGTTTGATGAGG -3' | *ARR2* | qPCR |
| ORE1 F | 5'-GCTACTGCCATTGGTGAAGT-3' | *ORE1* | qPCR |
| ORE1 R | 5'-TTCCGGTCTCTCACACAGAA-3' | *ORE1* | qPCR |
| EIN3 F | 5'- CCAGTGAACTCAGTAGCCCA -3' | *EIN3* | qPCR |
| EIN3 R | 5'- CTGTGGGTTGAAGCAGTGAC -3' | *EIN3* | qPCR |
| NYE1 F | 5'- AGCAGCAGCAGCTCACTCT -3' | *NYE1* | qPCR |
| NYE1 R | 5'- CCTAGGGAGCGTTGAAGGATG -3' | *NYE1* | qPCR |
| ACS2 F | 5'- TTGATCCGGAGAGGGTGGTT -3' | *ACS2* | qPCR |
| ACS2 R | 5'- ACCTGTCCGCCACCTCAAG -3' | *ACS2* | qPCR |
| ACT2 F | 5'- GGCTCCTCTTAACCCAAAGGC -3' | *actin* | qPCR |
| ACT2 R | 5'- CACACCATCACCAGAATCCAG -3' | *actin* | qPCR |
| AHP2 F | 5'- ATGGACGCTCTCATTGCTCAG -3' | *AHP2* | qPCR |
| AHP2 R | 5'- GCACACTAGCACCTACCTGAC -3' | *AHP2* | qPCR |
| AHP1 F | 5'- GCAGCTCCAGTATAGGAGCACA -3' | *AHP1* | qPCR |
| AHP1 R | 5'- CTCCAGCTTGAACAGAGTCTCT -3' | *AHP1* | qPCR |
| ARR1 F | 5'- AACGTAGCAAGTCACCTCCAG -3' | *ARR1* | qPCR |
| ARR1 R | 5'- GATTGCCTTGGTGCTGCGAT -3' | *ARR1* | qPCR |
| ARR10 F | 5'- GGCTTCTGATGCTGGTTCCTT -3' | *ARR10* | qPCR |
| ARR10 R | 5'- CCAATCACCTTCCGAGAAATCAG -3' | *ARR10* | qPCR |
| ARR12 F | 5'- ACGACGAACCAAGCACAAAA -3' | *ARR12* | qPCR |
| ARR12 R | 5'- TGGATCACTATGCGCAGACA -3' | *ARR12* | qPCR |
| AHK2 F | 5'- CTCTCCTCTCTGGCAAGGCA -3' | *AHK2* | qPCR |
| AHK2 R | 5'- CCTCATCTGCAAGGTGCACTG -3' | *AHK2* | qPCR |
| AHK4 F | 5'- ATCCGACTGTCGAAGAGCGT -3' | *AHK4* | qPCR |
| AHK4 R | 5'- GGTTACCAGCAAGCTGACCA -3' | *AHK4* | qPCR |
| CRF5 F | 5'- CTAAACCGGCGAGGAAAGCG -3' | *CRF5* | qPCR |
| CRF5 R | 5'- ATCACGAATCTCCGCCGCAA -3' | *CRF5* | qPCR |
| CRF6 F | 5'- TAGCGACGACGACGACAACA -3' | *CRF6* | qPCR |
| CRF6 R | 5'- CGCTTTCCTCCTCCTTGTGC -3' | *CRF6* | qPCR |
| ARR7 F | 5'- TCATCTGAGAACATCTTACCTCGT-3' | ARR7 | qPCR |
| ARR7 R | 5'- TTCACCGGTTTCAACAAGAAT -3' | ARR7 | qPCR |
